# Deep caries management: EFCD-ESE-ORCA S3-level clinical practice guideline

**DOI:** 10.1007/s00784-025-06727-1

**Published:** 2026-04-22

**Authors:** Falk Schwendicke, Esra Kosan, Avijit Banerjee, Aylin Baysan, Lars Bjørndal, Laura Ceballos, Henry F. Duncan, Sascha Herbst, Klaus W. Neuhaus, Anne C. O’Connell, Sebastian Paris, Helena Dujic

**Affiliations:** 1 Department of Conservative Dentistry, Periodontology and Digital Dentistry, LMU University Hospital, LMU Munich, Goethestraße 70, Munich, 80336 Germany; 2 Department of Periodontology, Oral Medicine and Oral Surgery, Charité – Universitaetsmedizin Berlin, Berlin, Germany; 3 Centre of Oral Clinical Translational Sciences/Conservative & MI Dentistry, Faculty of Dentistry, Oral & Craniofacial Sciences, King’s College London, London, UK; 4 Institute of Dentistry, Faculty of Medicine and Dentistry, Queen Mary University of London, London, UK; 5 Department of Odontology, Section of Clinical Oral Microbiology, Cariology and Endodontics, University of Copenhagen, Copenhagen, Denmark; 6 Area of Stomatology, Faculty of Health Sciences, Rey Juan Carlos University, Alcorcón, Madrid, Spain; 7 Division of Restorative Dentistry and Periodontology, Trinity College Dublin, Dublin Dental University Hospital, University of Dublin, Dublin, Ireland; 8 Department of Restorative, Preventive and Pediatric Dentistry, School of Dental Medicine, University of Bern, Bern, Switzerland; 9 Division of Public and Child Dental Health Dublin Dental University Hospital, Trinity College Dublin, University of Dublin, Dublin, Ireland; 10 Department of Operative, Preventive and Pediatric Dentistry, Charité – Universitaetsmedizin Berlin, Berlin, Germany; 11 Conservative Dentistry, Periodontology and Digital Dentistry, LMU University Hospital, LMU Munich, Goethestraße 70, Munich, 80336 Germany

**Keywords:** Clinical practice guideline, Deep caries, Direct pulp capping, Evidence-based dentistry, Pulpotomy, Selective excavation, Non selective caries removal, Stepwise caries removal, Vital pulp treatment

## Abstract

**Objective:**

To develop an evidence-based S3-level clinical practice guideline for the management of deep and extremely deep caries in vital permanent teeth.

**Methods:**

An evidence-based medical guideline based on systematically searched and appraised evidence as well as a structured consensus (S3-level) was jointly developed by the European Federation of Conservative Dentistry (EFCD), the European Society of Endodontology (ESE), the Organization for Caries Research (ORCA) and the German Society of Conservative Dentistry (DGZ), following the methodological framework of the Association of Scientific Medical Societies in Germany (AWMF) and the GRADE approach. Four working groups formulated key clinical questions regarding (1) caries removal strategies, (2) cavity liners, (3) management of exposed pulps and (4) materials for direct pulp capping and pulpotomy. Systematic reviews were conducted for each question and evidence was synthesized and graded for quality. A structured consensus process was used to formulate recommendations. In order to encourage its wide dissemination, this article is freely accessible on Clinical Oral Investigations 10.1007/s00784-025-06727-1, International Endodontic Journal 10.1111/iej.70132 and Caries Research 10.1159/000551659 journals’ websites.

**Results:**

Evidence supports selective (SE) or stepwise caries removal (SW) over non-selective removal (NSE) to reduce the risk of pulp exposure in deep caries. Routine use of cavity liners after caries removal showed no consistent clinical benefit and is not routinely recommended. For vital pulp therapy following pulp exposure, both direct pulp capping and pulpotomy are effective options in teeth without irreversible pulpitis, while pulpotomy is an acceptable alternative to pulpectomy in cases with signs of irreversible pulpitis. Hydraulic calcium silicate cements demonstrated superior clinical outcomes compared to calcium hydroxide and should be preferred for pulp capping and pulpotomy. The certainty of evidence ranged from very low to moderate across questions and outcomes.

**Conclusions:**

For deep caries, maintaining pulp vitality by using less invasive management strategies is supported by current evidence. Implementation of this guideline requires clinician training, patient-centered decision-making and consideration of economic and practical factors. Further research is needed, particularly for extremely deep caries and towards long-term outcomes.

**Supplementary Information:**

The online version contains supplementary material available at 10.1007/s00784-025-06727-1.

## Introduction

### Caries and deep and extremely deep caries lesions

#### Definition

Dental caries is a disease that originates from an ecological imbalance within the dental biofilm. Under healthy conditions, the biofilm comprises a balanced microbial community, with frequent intake of fermentable dietary carbohydrates shifting this equilibrium towards acid-producing (acidogenic), acid-tolerant (aciduric), and cariogenic microorganisms. This microbial shift alters the functional activity of the biofilm, tipping the balance between demineralization and remineralization in favor of mineral loss within the dental hard tissues [1].

Importantly, dental caries is not an infectious disease that requires eradication of bacteria, but a behaviorally-modifiable condition driven by biological, behavioral, psychosocial, and environmental factors—primarily the frequent consumption of fermentable carbohydrates and the persistence and maturation of dental biofilms [2]. Effective management focuses on modifying these causal factors through patient dietary management and oral hygiene practices (biofilm control). However, if such measures are not successfully implemented or followed, the ongoing metabolic activity of the biofilm can lead to an aggravation of the imbalance between demineralization and remineralization and, hence, lesion progression. At some point, early non-cavitated lesions show surface cavitation, which has been defined as important decision-point for intervening in the caries process [3]. Further progression can lead to “deep” or “extremely deep” caries lesions, potentially resulting in pulp inflammation, pulp necrosis, and apical periodontitis [4].

The terms “deep caries” and “extremely deep caries” have been defined by the European Society of Endodontology (ESE) as follows [5]: “Deep caries” extends into the inner quarter of the dentine but retains a protective layer of hard or firm dentine over the pulp, reducing the risk of immediate exposure; it is visible on radiographs when affecting proximal or occlusal surfaces. In contrast, “extremely deep caries” penetrates the entire dentine thickness, with no visible barrier to the pulp chamber making dental pulp exposure highly likely during treatment. Both conditions require careful and time-sensitive management to preserve dental pulp vitality when possible. The present guideline focuses on both types of caries, particularly those in teeth with vital pulps.

Note that for reasons for practicality, a simplified terminology was employed in this guideline (e.g. the term “carious” was not used). We also do not separate caries, the disease and process, from caries lesions, the signs of the disease. This was done as this guideline focuses on adoption by a wider clinical audience. It is acknowledged that for academic readers, a more detailed terminology may be more warranted. We outline the used terminology in the glossary of this guideline.

#### Prevalence

Recent epidemiological studies indicate that while global prevalence of untreated caries has remained consistently high over the past 25 years, there has been a demographic shift in the burden from children to adults [6, 7]. Dental caries remains the most widespread noncommunicable disease, with higher prevalence among socioeconomically disadvantaged groups [8–10]. Managing caries imposes significant financial strain, accounting for approximately 5% of total health expenditures in both industrialized and developing nations [11, 12].

Deep and extremely deep caries are common, affecting individuals of all age groups, though the prevalence varies by population, age, and socio-economic status. Studies indicate that children and young adults are particularly vulnerable. Overall, however, epidemiological data on deep and extremely deep caries are scarce, as detecting them, by large, hinges on further assessment, using mainly radiographs. A study from Sweden found that 22% of all 15-year olds suffered from deep restored caries [13], with the majority involving occlusal surfaces of first molars. Similarly, data from Norway found that one quarter of children exhibited deep restored or untreated caries. A significant proportion of these were associated with the tooth being root-canal treated or extracted as a result of the deep caries [14].

#### Treatment and consequences of failure to treat

Treatement of deep and extremely deep caries has shifted from eliminating all bacterially contaminated dentine to an emphasis on conserving tooth structure and avoiding pulp complications. Treatment options encompass different caries removal strategies, often combined with the management of the exposed pulp (vital pulp treatment, VPT) to more invasive interventions like pulpectomy (root canal treatment) or extraction in severe cases where maintaining pulp vitality/sensibility is no longer possible. If left untreated, deep and, more so, extremely deep caries can lead to pulp necrosis, periapical abscesses, systemic infections, and significant pain. Furthermore, these cases can be associated with higher treatment costs.

##### Caries removal

For teeth with vital pulps and no symptoms of irreversible pulpitis, four caries removal techniques are available, categorized based on the hardness of the remaining dentine [15, 16].

Non-Selective removal to Hard Dentine (NSE), previously known as complete caries removal, applies a uniform standard for removing decayed tissue across the cavity, ensuring that only hard dentine remains. This approach eliminates all demineralized dentine, even if bacteria-free.

Selective Removal (SE) to Firm Dentine leaves firm (leathery) dentine over the dental pulp while ensuring that peripheral dentine is hard. This technique, which maintains some resistance to hand excavation, has been recommended for deep caries (radiographically extending into the inner third of the dentine and potentially when caries extends to the inner quarter of the dentin) [17].

SE to Soft Dentine (formerly termed incomplete or partial removal) preserves soft caries tissue near the dental pulp to minimize exposure while ensuring that peripheral dentine is prepared to hard consistency for a secure restoration.

Stepwise Removal (SW) is a staged approach to caries removal, completed over two visits [18–20]. In the first stage, soft caries tissue is retained over the dental pulp, while the peripheral dentine is prepared to hard consistency to allow for a sealed restoration. A provisional restoration is placed, designed to last up to 12 months or longer, to enable dentine and pulp changes [21]. This initial phase encourages reactionary dentine formation, promotes remineralization, and decreases bacterial activity. During re-entry, the provisional restoration is removed, and changes in lesion color and hardness are reassessed. A standardized color classification scale can assist in evaluating cariogenic activity [19, 22–24]. Drier lesions often indicate reduced caries activity (Kidd et al. 1993). Caries removal continues only until firm dentine remains over the dental pulp. Some evidence suggests that in deep lesions, reopening the cavity comes with the risk of pulp exposure and potential harm [16, 21, 25]. Additionally, the second step requires extra cost, time, and potential discomfort for the patient.

Various methods have been proposed for assessing caries removal, including evaluating hardness, moisture content, color, fluorescence properties, and stainability with dyes [26]. Additionally, techniques such as self-limiting burs and chemo-mechanical removal have been developed to help define the endpoint of caries removal. While many of these techniques have been validated in laboratory settings, clinical validation remains limited [27]. All available methods for assessing the dentine remaining after caries removal serve as surrogate measures and should be validated against key clinical outcomes, including pain relief, maintenance of pulp health, and tooth retention.

##### Vital pulp treatment (VPT)

In deep and extremely deep caries, bacterial stimuli induce an inflammatory pulp response which progressively intensifies with caries advancement [28, 29]. The risk of bacterial presence in tertiary dentine and subjacent pulpal tissue is lower in deep caries as opposed to extremely deep caries [30].

Notably, the pulp possesses an intrinsic capacity for healing if the bacterial challenge is eliminated and the tooth is properly restored with a biocompatible material [31, 32]. Even for exposed pulps, successful management is possible due to advancements in biomaterials, techniques, and the understanding of pulp repair mechanisms at the cellular level. These management options include direct pulp capping [33], partial pulpotomy [34], and full pulpotomy [35]. Notably, even conventional direct pulp capping using calcium hydroxide has been found successful by single studies; the generalizability of these studies remains unclear, though [36].

If pulp exposure occurs in teeth with vital pulps and no symptoms or symptoms of reversible pulpitis, VPT is considered technically less complex compared to root canal treatment [37] and more cost-effective [38].

#### Economic aspects

The economic implications of deep and extremely deep caries are substantial, encompassing direct and indirect costs. Direct costs include expenses related to diagnostic procedures, treatment, and follow-up care, while indirect costs arise from lost productivity, absenteeism, and diminished quality of life. There is limited evidence on the economic impact of different strategies to address deep caries, while maintaining pulp vitality is likely to be cost-effective (as initial treatment costs, for example for endodontic therapy, are reduced, but mainly as fewer re-interventions are needed later on) [39].

For healthcare systems, untreated deep caries represents a considerable burden, often requiring emergency interventions and complex restorative procedures. In low-resource settings, the financial barriers to accessing dental care exacerbate disparities in oral health outcomes. Policymakers and stakeholders should consider the economic impact of deep caries to prioritize resources and develop sustainable models of care that emphasize prevention and accessibility.

### Clinical practice guideline aim

We aimed to develop an S3 guideline, with S3-level being the highest quality level of an evidence-based medical guideline in Germany. It is based on a systematic search and appraisal of the scientific evidence, as well as a structured consensus process among relevant professional societies. The guideline aimed to provide evidence-based recommendations for managing deep and extremely deep caries in the permanent dentition. Its goal is to improve dental care globally by reducing pain, infection, and impaired quality of life, ultimately aiming to prevent tooth loss.

### Patient target population

This guideline focuses on the management of deep and extremely deep caries in the permanent dentition in individuals of all ages.

### Target audience

The primary audience for this guideline includes general dentists and dental specialists, such as pediatric dentists and endodontists, as well as dental therapists or hygienists if they are involved in the diagnosis and treatment of deep and extremely deep caries.

### Exceptions from guideline

This guideline does not address the treatment of deep caries in primary teeth.

## Methodology

### General framework

This guideline was developed in alignment with the methodological framework provided by the Standing Guideline Commission of the Association of Scientific Medical Societies in Germany (AWMF) (https://www.awmf.org/leitlinien/awmf-regelwerk/awmf-guidance.html) [40] and the Grading of Recommendations Assessment, Development and Evaluation (GRADE) Working Group (https://www.gradeworkinggroup.org/). The project was conducted under the leadership of the European Federation of Conservative Dentistry (EFCD), the European Society of Endodontology (ESE), the Organization for Caries Research (ORCA) and the Deutsche Gesellschaft fuer Zahnerhaltung (DGZ). Oversight of the development process was provided by a Guideline Steering Group (GSG) and an independent methodology consultant (Ina Kopp, appointed by the societies). The GSG was constituted by Falk Schwendicke, Sebastian Paris, Aylin Baysan, Lars Bjørndal, Hal Duncan, Klaus Neuhaus, Esra Kosan and Helena Dujic. The guideline methodologist had no voting rights.

The GSG led discussions during guideline development meetings and at an online consensus summit. Initially, it was determined that the management of deep caries should be addressed by four Working Groups (WGs), each responsible for a specific clinical area: WG1: Caries removal – Chairs: Aylin Baysan and Lars Bjørndal. WG2: Cavity liners – Chairs: Esra Kosan and Sebastian Paris. WG3: Managing the exposed pulp – Chairs: Hal Duncan and Klaus Neuhaus. WG4: Materials used for direct capping or pulpotomy – Chairs: Falk Schwendicke and Sascha Herbst. The GSG met independently of the wider guideline panel to manage and monitor progress, with virtual meetings taking place regularly throughout 2023, 2024, and 2025. The Group identified and invited a range of scientific organisations, academic bodies, a student group, and other relevant stakeholders to participate in the guideline development process, and initiated a kick-off meeting in March 2025 (Supplementary Table 1).

Each organisation or stakeholder group was invited to nominate one representative to participate, provided they accepted the invitation (Supplementary Table 1). These representatives were subsequently invited to attend an online methodology workshop and the final consensus summit (Supplementary Table 2). In addition, invitations to participate in the guideline development process were extended to the European Patients’ Academy (EUPATI) and the German patient advocacy organization *BundesArbeitsGemeinschaft der Patientenstellen und -Initiativen* (BAGP); however, no response was received from the latter. National societies are invited to use this guideline for adaptation, adoption, and de novo development (adolopment) [41, 42].

### Evidence synthesis

In the initial phase of guideline development, a targeted search was conducted to identify existing clinical practice guidelines (CPGs) related to the management of deep caries. Notably, the ESE S3-level CPG on Treatment of Pulpal and Apical Disease was reviewed and considered [41]. Relevant recommendations from this guideline were integrated where appropriate, while the present guideline expands upon its scope by addressing additional clinical questions and incorporating the latest evidence. Specifically, four systematically developed PICOTS (Population, Intervention, Comparator, Outcome, Timing, Setting) questions, each addressing a clinically relevant decision in the management of deep or extremely deep caries in vital permanent teeth. These questions reflect core aspects of caries treatment and vital pulp therapy and were developed and agreed upon by the GSG and the WGs. The four focus points guiding the evidence review and recommendation process were:

1. Effectiveness of SW, SE compared to NSE removal in managing deep caries in vital permanent teeth.
2. Effectiveness of different cavity liners compared to no cavity lining in managing deep caries in vital permanent teeth.
3. Effectiveness of direct pulp capping compared to partial or full pulpotomy in managing deep caries in vital permanent teeth.
4. Effectiveness of calcium hydroxide compared to hydraulic calcium silicate cements (HCSCs) for direct pulp capping in managing deep caries in vital permanent teeth. This question was later expanded to also cover calcium hydroxide compared to HCSCs for pulpotomy, supported by a search for systematic reviews on this matter.

The four PICOTS questions were addressed through dedicated systematic reviews, each corresponding to a thematic focus of the working groups: (1) caries removal, (2) cavity lining, (3) management of the exposed pulp, and (4) materials for pulp capping or pulpotomy [43–46]. Table 1 provides an overview of the PICOTS elements for each question and their alignment with the respective systematic review.

**Table 1:** PICOTS questions addressed by each systematic review are listed according to working group: (1) caries removal, (2) cavity lining (3), management of the exposed pulp and (4) materials for pulp capping or pulpotomy

WG & Reference	Review title	Focussed PICOTS question
WG 1 Ramezanzade et al. 2025 [45]	Effectiveness of stepwise or selective in comparison to non-selective caries removal in managing deep caries in vital permanent teeth: A systematic review with trial sequential, pairwise and network meta-analyses	In patients with deep caries in mature or immature permanent teeth associated with no symptoms or those of a reversible pulpitis (P), is SW or SE (I) as effective as NSE (C), in terms of a combination of patient and clinical reported outcomes (O), with “tooth survival” as the most critical outcome?
WG 2 Kosan et al. 2025 [46]	Efficacy of different cavity liners compared to no cavity lining in managing deep caries in vital permanent teeth - A Systematic Review	In patients with deep caries in mature or immature permanent teeth associated with no symptoms or those of a reversible pulpitis (P), is a cavity lining of a certain material (I) as efficacious as other materials or no cavity lining (C), in terms of a combination of patient and clinical reported outcomes (O), with “failure” as the most critical outcome?
WG 3 Louzada et al. 2025 [44]	The effectiveness of pulpotomy (partial/full) compared with pulpectomy in managing deep caries in vital permanent teeth with a diagnosis of non-traumatic pulpitis	In patients with deep caries in mature or immature permanent teeth associated with no symptoms or those of a reversible pulpitis (P), is direct pulp capping (I) as effective as partial or full pulpotomy (C), in terms of a combination of patient and clinical reported outcomes (O), with “tooth survival” as the most critical outcome?
WG 4 Herbst et al. 2025 [43]	Effectiveness of calcium hydroxide compared to hydraulic calcium silicate cements for direct pulp capping in managing deep caries in vital permanent teeth: A systematic review and meta-analysis	In patients with deep caries in mature or immature permanent teeth associated with no symptoms or those of a reversible pulpitis (P), is direct pulp capping with Calcium Hydroxide (I) as effective as direct pulp capping with HCSCs (C), in terms of a combination of patient and clinical reported outcomes (O), with “tooth survival” as the most critical outcome? * *This question was later on expanded to also cover materials used for pulpotomy (see below).

For all four questions, T (Time) was defined as a minimum of 12 months and as long as possible for all outcomes, except for “pain, tenderness, swelling, and need for medication,” which required a minimum follow-up of 7 days and up to 3 months. For OHRQoL, a minimum follow-up of 6 months was specified. S (Setting) included randomized controlled trials (RCTs), controlled clinical trials (CCTs) and, where applicable, high-quality observational studies published since 1990. All systematic reviews followed the PRISMA guidelines [47] and were prospectively registered in PROSPERO.

#### Systematic searches

For each of the four key questions, literature searches were performed in PubMed (MEDLINE), Cochrane Library, and Ovid, using PICOTS-based strategies combining MeSH terms and free-text keywords with Boolean and proximity operators. Searches were limited to English-language studies involving humans and covered the period from 1990 to the end of 2024; an update search for 2024 was conducted in March 2025. Study selection followed a two-step process: independent screening of titles/abstracts by calibrated two reviewers, followed by full-text assessment against predefined eligibility criteria. Disagreements were resolved through discussion or third-party adjudication. Screening and selection were supported by standardised spreadsheets and tools such as Rayyan [48].

#### Outcomes

For each of the four PICOTS questions, a predefined set of outcomes was established before literature screening and data extraction. These outcomes captured clinically relevant effects, patient-centred endpoints, and potential complications, and were selected to inform decision-making in the management of deep and extremely deep caries in vital permanent teeth. The outcomes included:

- Failure: loss of sensitivity requiring endodontic treatment (direct pulp capping, partial or total pulpotomy, pulpectomy) or restorative retreatment (restoration fracture or retention loss) or extraction.
- Loss of pulp vitality, risk of pulpal complications, including pain, tenderness, swelling.
- Patient-reported outcomes, including oral health-related quality of life (OHRQoL) and treatment burden.
- Restoration longevity.
- Adverse events.
- Economic aspects (where available).

Outcome prioritisation was informed by a 9-point GRADE Likert scale. A structured questionnaire was completed by a patient representative to rate the importance of each outcome from a patient perspective. Outcomes rated as critical (scores 7–9) were prioritised in the evidence synthesis and summary of findings.

#### Data synthesis

All data were analysed qualitatively and quantitatively and a narrative synthesis of the included studies was performed. If the included studies were homogeneous in nature, a quantitative meta-analysis was considered. The software used to perform a potential meta-analysis was determined by the review team based on previous experience and availability in respective review centres.

#### Critical appraisal and quality assessment

The risk of bias assessment was carried out according to the literature available. For RCTs, the Cochrane Risk of Bias tool (RoB) was chosen, which covered the domains of sequence generation, concealment of allocation, participant and personnel blinding, completeness and transparency of outcome reporting and the handling of incomplete data [49]. The overall risk of bias was classified in three categories: Low risk of bias - The study is well-conducted with minimal concerns about bias. Some concerns - There is uncertainty due to missing information or methodological weaknesses that may introduce bias. High risk of bias -The study has significant flaws that are likely to distort the results.

For CCTs the risk of bias was assessed using the Risk Of Bias In Non-randomized Studies of Interventions (ROBINS-I) tool which evaluates bias across domains including confounding, selection of participants, classification of interventions, deviations from intended interventions, missing data, measurement of outcomes and selection of the reported result. The risk of bias was classified as: Low risk of bias -The study is comparable to a well-performed randomized trial. It is judged to be at low risk of bias across all domains. Moderate risk of bias - The study is sound for a non-randomized study but cannot be considered comparable to a well-performed randomized trial. Serious risk of bias - The study has important problems in at least one domain that put it at serious risk of bias. Critical risk of bias - The study has problems in one or more domains of sufficient severity that no confidence can be placed in the estimate of effect. No information - There is insufficient information to permit a judgment.

The certainty of evidence for each key question and outcome was assessed using the GRADE approach [50]. For each outcome, the following GRADE domains were assessed: risk of bias, inconsistency, indirectness, imprecision, and publication bias. The overall certainty of the evidence was categorised as high, moderate, low, or very low. Where necessary, expert judgement was applied to address issues such as incomplete reporting, indirectness, or methodological uncertainty.

#### Evidence to recommendations

The evidence-based recommendations and statements were drafted based on the results of the systematic reviews, the additional search to cover different materials for pulpotomy in question 4, and by considering the balance of benefits and harms, the quality of evidence, the importance of patient-relevant outcomes, and the anticipated impact on clinical practice. Patient input from outcome prioritisation was also taken into account. Our process was aligned with the Evidence-to-Decision (EtD) framework recommended in the AWMF Guidance Manual [40]. To categorise the strength of recommendations, a three-stage wording scheme was used as shown in Table 2.

**Table 2:** Strength of recommendations: grading scheme by the AWMF

Grade of Recommendation	Description	Phrasing
A	Strong recommendation	We recommend (⇑⇑)
		We recommend not to (⇓⇓)
B	Weak recommendation	We suggest (⇑)
		We suggest not to (⇓)
0	Recommendation open	May be considered (⇔)

For all evidenced-based recommendations and statements, this guideline highlights: (1) the ‘quality of evidence’ available to support each specific outcome, an evaluation that reflects the degree of certainty or uncertainty of the evidence; (2) the ‘grade of the recommendation’, reflecting the criteria considered to make the judgement; the strength of consensus and the percentage number of abstentions due to potential conflicts of interest.

#### Structured consensus

All recommendations and statements were subjected to a structured consensus process. A formal online consensus conference was scheduled on 12 June 2025 and conducted under the guidance of the independent moderator. The consensus determination process followed the recommendations of the AWMF and its Standing Guidelines Commission [40]. Strong consensus (agreement > 95%) or consensus (agreement 75–95%) was reached on all items. Participants with an agreed conflict of interest, who were not permitted to vote, were excluded from the consensus calculations.

#### Independence

The development of this guideline and its subsequent publication was financed entirely by funds of organizing societies, without any support from industry, other organizations or stakeholders. Potential conflicts of interest were addressed throughout the guideline process, including in WG meetings and the consensus session. All participants submitted declarations of interest via the AWMF online portal using a standardised form (version 2018) prior to the consensus phase. Declarations from the guideline coordinators were reviewed by independent assessors; all others were assessed by the coordinating team. Interests were categorised by their potential relevance, and individuals with conflicts abstained from voting. Declared interests were presented at the consensus summit, and measures such as independent moderation and a multidisciplinary panel composition were implemented.

#### Validity and update process

This guideline becomes valid upon publication and is intended to remain in effect for a period of five years, unless new, practice-changing evidence becomes available earlier. The need for an update will be assessed no later than 2030.

## Clinical recommendations for managing deep caries

Managing deep caries can involve a sequence of events; from caries removal, to possible placement of cavity lining and restoring the cavity. If the dental pulp exposure occurs, managing it using different strategies and materials may be required. The four WGs reflected these different steps, and the recommendations below are guiding practitioners along the possible therapeutic pathways:

- For caries removal, the different strategies have been reported. As our evidence syntheses and recommendations will reflect, these strategies come with different risks of complications, namely pulp exposure (which is widely reported in the literature), pulpal and restorative failures, up to tooth loss. Pulp exposure itself will need to be addressed by subsequent management, as reported in the third set of recommendations. The risk of failure (and involved therapeutic efforts) of pulp management are key aspects of how to decide during caries removal: As shown later, for some (specialized) practitioners and under some settings or for some patients, managing the exposed pulp would be highly successful, so exposing the pulp is a risk of lower relevance. For the majority of dentists, settings and patients, however, the management of the exposed pulp would be a challenge – technically and financially. Our recommendations reflect this and are a call for considerate decision-making. The latter is particularly true for extremely deep caries, which comes with potential specific challenges. Further research is required towards how to best approach these lesions.
- For cavity lining, the effort of placing a liner/pulp protection should be weighed against the involved efforts and costs. The decision-making in extremely deep caries is, again, challenging given the absence of any evidence beyond anecdotes. Our recommendations are specifically addressing routine situations, with extremely deep caries likely being “routine”.
- For the exposed pulp, direct capping or pulpectomy have long been the standard management procedures. As laid out, recent evidence indicates that pulpotomies may also be successful for permanent teeth in this indication. Our recommendations reflect this, and highlight once more that clinicians should weigh the application of different VPT strategies against the involved costs, efforts, and patients’ preferences. For teeth with pulps showing signs of irreversible pulpitis, pulpotomy may also be chosen as a means to avoid the more invasive pulpectomy, which can be regarded as the current standard for this indication. Our recommendations are a call to action for solidifying the evidence in this direction, but also for considering a less invasive approach when managing irreversible pulpitis.
- For choosing the material used for capping the exposed or pulpotomized pulp, our review found clear evidence supporting one material, with associated cost and handling issues.

### Stepwise or Selective Caries Removal versus Non-Selective Caries Removal

In patients with deep caries in mature or immature permanent teeth associated with no symptoms or those with reversible pulpitis (P), is SW or SE (I) as effective as NSE (C), in terms of a combination of patient and clinical reported outcomes (O), with “tooth survival” as the most critical outcome?

(T) = Defined as minimum of 12 months and maximum of as long as possible for all outcome measures, except pain, tenderness, swelling, need for medication (analgesics), where T was a minimum of seven days and maximum of three months, and OHRQoL, where T was minimum of six months and maximum of as long as possible.

#### Recommendations

**Table:** 

R1.1	Evidence-based recommendation	New Status (2025)
***Grade of recommendation***: B (⇑)	*In patients with deep caries in permanent teeth associated with no symptoms or symptoms indicative of reversible pulpitis* • **we suggest** selective removal to soft dentine (SE) as opposed to non-selective removal to hard dentine (NSE) (formerly complete removal).	
***Quality of evidence***:	***Supporting literature*** • Ramezanzade et al. 2025 (systematic review) with five randomized trials (RCTs) (*N*= 379) [45].	
Failure (SE and NSE): Very low ⊕⊝⊝⊝	• Three (RCTs) (Ahmed et al. 2021; Khokhar and Tewari 2018; Orhan et al. 2010) (*N*= 256) [23, 51, 52].	
Loss of pulp vitality (SE and NSE): Very low ⊕⊝⊝⊝	• Two RCTs (Khokhar and Tewari 2018; Rando-Meirelles et al. 2012) (*N*= 152) [52, 53].	
Postoperative pain (SE and NSE): Very low ⊕⊝⊝⊝	• Two RCTs (Duman et al. 2021; Rando-Meirelles et al. 2012) (*N*= 76) [26, 53].	
Tooth survival not reported		
***Strength of consensus***	12 yes/0 no/0 abstention; 100% consensus Strong consensus	

**Table:** 

R1.2	Evidence-based recommendation	New Status (2025)
***Grade of recommendation***: B (⇑)	*In patients with deep caries in permanent teeth associated with no symptoms*,* or symptoms indicative of reversible pulpitis* • **we suggest** stepwise removal (SW) as opposed to non-selective removal to hard dentine (NSE) (formerly complete removal).	
***Quality of evidence***:	***Supporting literature*** • Ramezanzade et al. 2025 (systematic review): four RCTs and one non-RCT (*N*= 692) [45].	
Failure (SW and NSE): Very low ⊕⊝⊝⊝	• Four RCTs (Orhan et al. 2010; Bjørndal et al. 2010; Leksell et al. 1996; Manhas et al. 2020) (*N* = 478.) [22, 23, 54, 55]	
Loss of pulp vitality (SW and NSE): Low ⊕⊕⊝⊝	• One RCT (Bjørndal et al. 2010; 2017) (*N*= 292) [22, 56].	
Postoperative pain (SW and NSE): Low ⊕⊕⊝⊝	• One RCT (Bjørndal et al. 2010; 2017) (*N*= 292) [22, 56].	
Restorative failure (SE and NSE): Very low ⊕⊝⊝⊝	• One RCT (Labib et al. 2019) (*N*= 126) [57].	
Tooth survival not reported		
***Strength of consensus***	13 yes/0 no/0 abstention; 100% consensus Strong consensus	

**Table:** 

S1.3	Evidence-based statement	New Status (2025)
*In patients with deep caries in permanent teeth associated with no symptoms or symptoms indicative of reversible pulpitis*,* the risk of dental pulp exposures can be reduced when performing selective removal to soft dentine (SE) or stepwise removal (SW) as opposed to non-selective removal to hard dentine (NSE) (formerly complete removal).*		
***Level of evidence***: Pulp exposure (SE and NSE): Low ⊕⊕⊝⊝ Pulp exposure (SW and NSE): Very low ⊕⊝⊝⊝	***Supporting literature***	
	• Ramezanzade et al. 2025 (systematic review) (*N*= 731) [45].	
***Strength of consensus***	12 yes/0 no/0 abstention; 100% consensus Strong consensus (statement)	

**Table:** 

S1.4	Expert consensus-based recommendation	New Status (2025)
*In extremely deep caries*,* the risk of pulp exposure is elevated and should be anticipated (see further recommendations). Further research is needed regarding the role of SE and SW in patients with extremely deep caries in permanent teeth associated with no symptoms*,* or symptoms indicative of reversible pulpitis*.		
***Supporting literature*** • Non-RCT (Oz et al. 2019) (*N*= 214) [58].		
***Strength of consensus***	12 yes/0 no/1 abstention; 92.3% consensus Consensus	

#### Background text

##### Intervention

Removal of caries is the intervention of choice when non-operative approaches (including non-invasive ones such as fluoride varnish application and micro-invasive ones such as sealants) are no longer indicated; this is usually the case for active cavitated caries. Several removal strategies are available:

- Selective Removal (SE) to either soft or firm dentine.
- Non-Selective Removal (NSE) to a hard or ‘sound’ dentine, formerly termed complete removal.
- Stepwise Removal (SW), a two-visit caries removal procedure starting with SE at pulpo-proximal surfaces, and NSE at peripheral surfaces, followed by a temporary restoration, and a second visit later on where SE to firm dentine is performed in pulpal areas and the definitive restoration.

##### Available evidence

Guideline recommendations are supported by a recent systematic review [45]. Six RCTs and one cohort study compared SE to NSE for the outcome of overall (any) failure (*n* = 379) [23, 26, 51–53, 59]; four RCTs compared them for the risk of pulp exposure. Four studies compared SW versus NSE [22, 23, 54–56] (*n* = 478) for the outcome of failure and for the risk of pulp exposure. Four RCTs and one non-RCT compared SW to NSE for the outcome of failure (*n* = 692) [22, 23, 54, 55, 58] and four RCTs compared them for the risk of pulp exposure (*n* = 531) [22, 23, 54, 55]. Three RCTs [23, 51, 52] and one retrospective observational study [60] compared SE versus NSE (*n* = 731 teeth). The certainty of the evidence underlying the recommendations was evaluated according to the GRADE methodology [50].

Based on this evidence, we suggest SE or SW instead of NSE in deep caries. Whether SE versus SW should be performed is not fully clear and can best be decided based on patient and dentist-related factors (preferences, efforts, availability) as well as caries penetration depth. For caries radiographically extending into the inner third, but not inner quarter of the dentine, SE may be preferred, while for those extending into the inner quarter of the dentine SW may also be employed. Notably, there is uncertainty around how to deal with extremely deep caries, i.e. those where on a radiograph, no radio-opaque band of residual dentine above the pulp is detectable. The latter finding, however, depends on the projection of the radiograph and is likely not reliable. Where this band is not detectable, a higher risk of pulp exposure may be present. Given the uncertainty in assessing this, our recommendation does not change when the band is detectable versus when it is not detectable. We do, however, recommend specific preparation for the possible pulp exposure and the subsequent management of the exposed pulp (see below).

The available evidence does not consistently distinguish between mature and immature permanent teeth in evaluating outcomes of caries removal strategies. As a result, it remains unclear whether root development stage influences treatment effectiveness or prognosis.

##### Risk of bias

Most studies had some concerns due to inconsistent reporting of blinding and allocation methods. Only few studies achieved a low overall risk of bias. No conflicts of interest were reported.

##### Concistency of study results

Despite methodological variability, the outcomes across studies were largely consistent. Significant heterogeneity in how failure, a composite measure indicating that the need for any kind of retreatment, was defined, was observed.

##### Balance of benefit and harm

No serious adverse effects were reported.

##### Ethical Considerations

No ethical concerns were raised in the included studies.

##### Accessibility, affordability and equity issues

A two-visit caries removal procedure may involve additional costs and come with a certain risk of patients not attending the second visit.

##### Legal considerations

None reported. In some healthcare systems, remuneration regulation requires NSE to be performed. This review is a call to action to update such regulations, accommodating the evidence.

##### Economic considerations

Economic benefits may arise if SE is performed as opposed to NSE or SW [39].

##### Patient preferences and values

Relatively low postoperative discomfort and high survival rates were reported for all interventions, indicating general patient acceptance of both approaches. This is likely influenced by the fact that the treatment aims to avoid root canal therapy. SE has been shown high acceptance by patients [61]. It should be noted that the composite outcome measure “failure” summarizes a range of clinically relevant outcomes, with individual patients weighing these outcomes differently. Future studies should aim to report the different clinical outcomes separately and comprehensively. Moreover, it should be noted that the outcome of tooth survival was not reported in the studies. This outcome, however, may be most relevant to patients.

##### Applicability

The evidence, derived from both university settings and general dental practice environments, supports broad applicability for all three caries removal strategies. It should be noted, however, that management of failures like pulp exposure may come with challenges in certain settings or for certain practitioners. Moreover, this management requires specific equipment (e.g. application of rubber dam, magnification) and materials, which may be costly (see below for further discussion). Aspects like operator expertise and experience, available equipment and materials, and affordability and patients’ willingness to pay should be considered when deciding for or against a certain strategy, with concious evaluation of the involved risks and potential needs for subsequent therapy.

### Cavity lining/pulp protection

In patients with deep caries in mature or immature vital permanent teeth associated with no symptoms or those of a reversible pulpitis (P), is a cavity lining of a certain material (I) as efficacious as other materials or no cavity lining (C), in terms of a combination of patient and clinical reported outcomes (O), with “failure” as the most critical outcome?

(T) = Defined as a minimum of 12 months and maximum of as long as possible for all outcome measures, except for ‘pain, tenderness, swelling, need for medication (analgesics), where T was a minimum of 7 days and maximum of 3 months, and Oral Health-Related Quality of Life (OHRQoL), where T was a minimum of 6 months and maximum of as long as possible.

#### Recommendation

**Table:** 

R2	Evidence-based recommendation	New Status (2025)
***Grade of recommendation***: B (⇑)	*In patients with deep caries caries in permanent teeth associated with no symptoms or symptoms indicative of reversible pulpitis* • **we suggest** not to routinely apply a cavity liner. *Further research is needed on the impact of residual dentine thickness after caries removal for decision making on whether to use or not to use cavity liners.*	
***Quality of evidence***: Failure: Very Low ⊕⊝⊝⊝ Tooth Survival: Low ⊕⊕⊝⊝ Postoperative Hypersensitivity: Very Low ⊕⊝⊝⊝ Secondary Caries: Low ⊕⊕⊝⊝ Restoration Longevity: Low ⊕⊕⊝⊝	***Supporting literature***	
	• Kosan et al. 2025 (systematic review): 12 RCTs (*n* = 1184). No Meta-analysis.	
***Strength of consensus***	13 yes/0 no/0 abstention; 100% consensus Strong consensus	

##### Background text

Cavity liners are traditionally applied after removing deep caries to protect the pulp, reduce postoperative sensitivity, and enhance the longevity of restorations.

##### Intervention

Cavity liners include calcium hydroxide-based materials, resin-modified calcium silicates, glass-ionomer cements, hydraulic calcium silcate cements and flowable composite liners, as well as resin-based ion-releasing liners or desensitizing polycarboxylate cements. Despite their widespread use, the clinical benefits of using a liner or no liner remain debated.

##### Available evidence

The systematic review analysed 12 RCTs involving 1184 teeth. The findings revealed no significant difference in failure between lined and unlined restorations. Absence of failure (no need for root-canal treatment or restorative retreatment) was reported at above 96%. Tooth survival across all groups was reported between 94% and 100%. Use of calcium hydroxide, glass-ionomer cements and resin-modified calcium silicate materials showed similar efficacy in preventing secondary caries and maintaining pulp vitality. Pain levels and postoperative hypersensitivity were generally low across studies, with no consistent benefit from liner use. The certainty of the evidence underlying the recommendations was evaluated according to the GRADE methodology.

In summary, the current evidence showed no consistent clinical advantage of a cavity liner in lowering the risk of failure, maintaining teeth and pulp vitality, or minimizing postoperative hypersensitivity when compared to not placing a liner. Considering this, and given the additional costs and procedural complexity, routine use of cavity liners cannot be generally recommended. The decision for or against a liner should be made based on clinical judgment, individual patient conditions, and economic considerations.

One aspect which is occasionally mentioned when discussing the need for a liner is the residual dentine thickness covering the pulp. Based on this anecdotal evidence, cavity floors in close relationship to the pulp (e.g. where the pulp may be visually detectable, shining through the dentine) should receive a liner. There is no evidence supporting this approach, though, and it is unclear if there is any such threshold indicating to use (or not use) a liner. Moreover, there is no reliable measure to assess the true residual dentine thickness.

The available evidence does not consistently distinguish between mature and immature permanent teeth in evaluating the effectiveness of different cavity lining materials or the omission of lining. As a result, it remains unclear whether root development stage influences treatment outcomes.

##### Risk of bias

Most studies had some concerns due to inconsistent reporting of blinding and allocation methods. Only a few studies achieved a low overall risk of bias. No major conflicts of interest were reported.

##### Consistency of results

Despite methodological variability, the outcomes across studies were largely consistent. No significant heterogeneity in failure or other outcomes was observed.

##### Balance of benefit and harm

No serious adverse effects were reported in either lined or unlined groups.

##### Ethical considerations

No ethical concerns were raised in the included trials.

##### Accessibility, affordability, and equity issues

While some liner materials may involve additional costs, the lack of clear clinical advantage questions their routine use, especially in low-resource settings.

##### Legal considerations

None reported.

##### Economic considerations

Economic benefits may arise from omitting liners when they offer no proven clinical advantage.

##### Patient preferences and values

Minimal postoperative discomfort and high tooth survival and success rates were reported regardless of liner use, indicating general patient acceptance of both approaches.

##### Applicability

The evidence, although derived from varied clinical settings, supports broad applicability. However, limitations in external validity exist due to short follow-up periods and controlled trial environments.

### Managing the exposed pulp

#### Research question

oIn patients with deep and extremely deep caries in mature or immature permanent teeth associated with no symptoms or those of a reversible pulpitis (P), is direct pulp capping (I) as effective as partial or full pulpotomy (C) for managing the exposed pulp, in terms of a combination of patient and clinical reported outcomes (O), with “tooth survival” as the most critical outcome?

(T) = Defined as minimum of 12 months and maximum of as long as possible for all outcome measures, except `pain, tenderness, swelling, need for medication (analgesics), for which T was a minimum of 7 days and maximum of 3 months, and OHRQoL, for which T was a minimum of 6 months and maximum of as long as possible.

#### Recommendation

**Table:** 

R3.1	Evidence-based recommendation updated from ESE S3-level recommendation	New Status (2025)
***Grade of recommendation***: B (⇑)	*In patients where a pulp is exposed during caries removal in permanent teeth associated with no symptoms or signs indicative of reversible pulpitis* • **we suggest** either direct pulp capping or pulpotomy (partial/full) after non-selective excavation (complete caries removal).	
***Quality of evidence***: Post-operative pain: Very low ⊕⊝⊝⊝ Clinical signs and symptoms and/or evidence of radiographic radiolucency: Very low ⊕⊝⊝⊝	***Supporting literature***	
	• Jakovljevic et al. 2023 (systematic review including 2 studies) [62]; ESE S3 guideline (Duncan et al. 2023) [41] o 1 st RCT (*n* = 218) o 2nd RCT (*n* = 276) Other outcomes including survival were not reported.	
***Strength of consensus***	13 yes/0 no/0 abstention; 100% consensus Strong consensus	

#### Research question

In patients with deep and extremely caries in permanent teeth associated with no symptoms, reversible pulpitis or signs and symptoms indicative of irreversible pulpitis (P), is partial pulpotomy (I) as effective as full pulpotomy (C) for managing the exposed pulp, in terms of a combination of patient and clinical reported outcomes (O), with “tooth survival” as the most critical outcome?

##### Recommendations

**Table:** 

R3.2a	Evidence-based recommendation	New Status (2025)
***Grade of recommendation***: B (⇑)	*In patients where a pulp is exposed during caries removal in permanent teeth associated with signs and symptoms indicative of irreversible pulpitis* • **we suggest** either partial or full pulpotomy after non-selective excavation (complete caries removal).	
***Quality of evidence***: Post-operative pain: Low ⊕⊕⊝⊝ Clinical signs and symptoms and/or evidence of radiographic radiolucency: Low ⊕⊕⊝⊝	***Supporting literature***	
	• Systematic review [44] and three RCTs [63–65]. Both outcomes: o 1 st RCT (*n* = 50) o 2nd RCT (*n* = 93) o 3rd RCT (*n* = 200) Other outcomes including survival were not directly reported.	
Strength of consensus	13 yes/0 no/0 abstention; 100% Strong consensus	

**Table:** 

R3.2b	Expert-based recommendation updated from ESE S3-level recommendation	New Status (2025)
**Expert consensus**	*In patients with a pulp exposed during caries removal*,* if direct pulp capping or pulpotomy (partial/full) are performed*, • **we suggest** an enhanced protocol (i.e., dental dam, intraoperative antimicrobial lavage, magnification and a suitable material).	
***Supporting literature*** Expert consensus, ESE guideline [5, 41] and published studies [44, 66].		
***Strength of consensus***	13 yes/0 no/0 abstention; 100% consensus Strong consensus	

#### Research question

In patients with non-traumatic pulpitis associated with no or nonspontaneous pain in mature permanent teeth (P), is pulpotomy (partial/full) (I) as effective as a pulpectomy (C) for managing the exposed pulp, in terms of a combination of patient and clinical reported outcomes (O), with “tooth survival” as the most critical outcome?

##### Recommendation

**Table:** 

S3.3	Evidence-based statement updated from ESE S3-level recommendation	New Status (2025)
*In patients with a pulp exposed during caries removal in permanent teeth*,* evidence suggests that partial or full pulpotomy seem to be as effective as pulpectomy (root canal treatment) in terms of outcomes.*		
***Level of evidence***: Post-operative pain: Low ⊕⊕⊝⊝ Clinical signs and symptoms and/or radiographic radiolucency: Low ⊕⊕⊝⊝	***Supporting literature***	
	• Supporting literature [62]. 1 study. ESE S3 guideline [41]. o 1 RCT (*n* = 54). Other outcomes not reported [44, 65].	
***Strength of consensus***	12 yes/0 no/1 abstention; 92.3% consensus Consensus	

### Background text

#### Intervention

Treatment of patients with deep caries and the exposed pulp can involve pulp capping, pulpotomy (partial or full) as well as pulpectomy. Each treatment has a different range of invasiveness and its choice is often based on pulp diagnosis, which is generally based on patients’ signs and symptoms [5, 67].

#### Available evidence

Part of the PICOT question has been addressed by the ESE S3-level CPG [41]. This guideline was incorporated into the recommendations. For the new PICOT 3.2a, the three selected studies included 343 teeth that qualified for the systematic review. All three studies were RCTs with overall good or moderate quality. Relative to outcomes, three studies addressed the patient-centred outcome postoperative pain and also all three addressed periapical healing. The certainty of the evidence underlying the recommendations was evaluated according to the GRADE methodology.

Based on this, we suggest to perform VPT – without providing any recommendation towards direct capping or different pulpotomies – for teeth with no or reversible pulpitis. For those with irreversible pulpitis, pulpotomies can be performed instead of pulpectomy, while clinically, this decision will be made based on further clinical signs during the procedure (hemostasis, pulp condition). Notably, pulpotomy is less invasive and likely also less costly than pulpectomy (see below). In any case, during VPT, an enhanced management protocol should be employed (i.e., dental dam, intraoperative antimicrobial lavage, magnification and a suitable material), ensuring high success rates. If such protocol is not employed, success may be significantly lower.

The available evidence does not consistently distinguish between mature and immature permanent teeth in evaluating outcomes of direct pulp capping compared to partial or full pulpotomy. Therefore, it remains unclear whether root development stage affects the success of VPT in cases of deep or extremely deep caries.

#### Risk of bias

Two of the RCTs were judged as overall of good quality, although one was limited by smaller numbers. The third RCT had a moderate risk of bias and was of low quality. Main heterogeneity issues concerned patient selection bias (mean age was 25 years), tooth selection bias (mostly lower molars were included, primary caries only), assessment of pulpitis, assessment of pain.

#### Concistency of study results

Patient-reported outcomes were inconsistently reported. Adverse events, when reported, were rare. Heterogeneity was moderate to high.

#### Clinical relevance of outcomes and effect size

- Radiographic healing: Current evidence suggests no difference between full pulpotomy versus partial pulpotomy. Notably, one study found higher success for full versus partial pulpotomy.
- Pain: Current evidence suggests that there was no difference in post-operative pain between partial and full pulpotomy in teeth with signs and symptoms indicative of irreversible pulpitis after one week. Pain relief was faster with full pulpotomy.

#### Balance of benefit and harm

The majority of the studies did not report on potential harm/adverse effects.

#### Ethical Considerations

None reported.

#### Accessibility, affordability and equity issues

Pulpotomies may require additional time and equipment (magnification, e.g. operation microscope), and may hence not be available in all practices, compared with direct pulp capping. Full pulpotomy may be more complex than partial pulpotomy, with unclear impact on accessibility and affordability.

#### Legal considerations

None reported.

#### Economic considerations

Pulpotomies may require additional time and equipment, and are hence likely more costly than direct pulp capping. Full pulpotomy may be more complex than partial pulpotomy, with unclear impact from an economic perspective.

#### Patient preferences and values

None reported.

#### Applicability

The majority of studies were conducted in well-controlled research environments and included specifically selected populations. Generalizability to general dental practice settings remains unclear, particularly as not all dentists will be able to provide the required isolation, equipment (magnification, instrumentation) and materials. Moreover, it can be assumed that not all dentists will be able to perform pulpotomies in permanent teeth given that pulpotomies in permanent teeth are not part of routine undergraduate training in most areas worldwide.

### Calcium Hydroxide compared to Hydraulic Calcium Silicate Cements for direct pulp capping and pulpotomy

In patients with deep caries in mature or immature permanent teeth associated with no symptoms or those of a reversible pulpitis (P), is direct pulp capping or pulpotomy with calcium hydroxide (I) as effective as direct pulp capping or pulpotomy with hydraulic calcium silicate cements (HCSC) (C), in terms of a combination of patient and clinical reported outcomes (O), with “tooth survival” as the most critical outcome?

(T) = Defined as minimum of 12 months and maximum of as long as possible for all outcome measures, except `pain, tenderness, swelling, need for medication (analgesics), for which T was was a minimum of 7 days and maximum of 3 months, and OHRQoL, for which T was minimum of 6 months and maximum of as long as possible.

#### Recommendation

**Table:** 

R4	Evidence-based recommendation	New Status (2025)
***Grade of recommendation***: B (⇑)	*In patients where a pulp is exposed during caries removal in permanent teeth with no symptoms or those of a reversible pulpitis*, • **we suggest** performing direct pulp capping or pulpotomy using a hydraulic calcium silicate cement instead of using calcium hydroxide.	
***Quality of evidence*** Absence of clinical symptoms and radiographic pathology: Moderate ⊕⊕⊕⊝ Postoperative pain (1-7d post intervention) Very low ⊕⊝⊝⊝	***Supporting literature***	
	• Herbst et al. 2025 (systematic review) [43] • Li et al. 2024 (systematic review) [68] • Absence of clinical symptoms and radiographic pathology: Information from 5 randomized-controlled trials based on teeth (*n* = 552). • Postoperative pain: Information from 2 randomized-controlled trials based on teeth (*n* = 121).	
***Strength of consensus***	12 yes/0 no/0 abstention, 100% consensus Strong consensus	

### Background text

#### Intervention

Direct pulp capping is the least minimal procedure for treatment of the cariously exposed pulp in teeth with no pulpitits or symptoms of reversible pulpitis. Pulpotomy, involving partial or complete removal of the coronal pulp, has increasingly been adopted as a treatment for permanent teeth with carious pulp exposure, including those with irreversible pulpitis. While calcium hydroxide has been used for decades, its drawbacks, such as local inflammation and poor mechanical stability, led to the development of hydraulic calcium silicate-based cements (HCSC), which offer improved mechanical properties and biological effects but may have disadvantages like tooth discoloration, handling challenges, and higher costs.

#### Available evidence

Systematic review by Herbst et al. 2025 for direct pulp capping: Five RCTs comprising 552 teeth with either 12 or 36 months follow-up 43. Clinical and radiographic absence of pathology: HCSC showed significantly higher probability for absence of pathology compared to calcium hydroxide (Odds Ratio (OR): 2.68, 95% confidence interval [1.70, 4.22]). Two studies evaluated postoperative pain using different metrics, with one study finding HCSC to reduce post-operative pain, and one study finding no difference between different DPC materials in post-operative pain. The certainty of the evidence underlying the recommendations was evaluated according to the GRADE methodology.

Systematic review by Li et al. 2024 for pulpotomy: Twenty-five RCTs including permanent teeth (*n* = 2012) with carious pulp exposure with ≥ 12 months follow-up assessed the overall success of pulpotomy, defined as absence of clinical symptoms and radiographic pathology [68]. The pooled success rate was 86.7% (95% CI: 82.0–90.7%). Success was lower in teeth with irreversible pulpitis (82.4%) than in those with normal pulp/reversible pulpitis (92.0%). Seven RCTs compared mineral trioxide aggregate (MTA) (195 teeth) to calcium hydroxide (182 teeth), showing higher success for MTA (OR: 2.41, 95% CI: 1.28–4.51). Six RCTs compared MTA (180 teeth) to Biodentine (168 teeth), showing no significant difference.

The available evidence does not consistently distinguish between mature and immature permanent teeth when comparing calcium hydroxide and hydraulic calcium silicate cements (HCSCs) for direct pulp capping or pulpotomy. Consequently, it remains uncertain whether root development stage influences the effectiveness of these pulp treatment materials.

#### Available evidence

For direct capping, one study was considered to have a high quality and a low risk of bias (*n* = 56 teeth); three studies showed some concerns (*n* = 427 teeth); one study was considered to have a high risk of bias (*n* = 69 teeth). No conflicts of interest were observed among the studies. Overall moderate certainty of evidence [43].

#### Risk of bias

For pulpotomy, most studies had moderate to high risk of bias, with only two trials rated as low risk across all domains. No conflicts of interest were reported [68].

#### Consistency of study results

In Herbst et al. 2025, despite a limited number of studies and a potentially biased I², overlapping confidence intervals for each effect size and low heterogeneity indicated consistency [43]. All studies were directly relevant, with no issues of indirectness; no industry sponsorship was reported.

In Li et al. 68, substantial heterogeneity was observed in the overall analysis of pulpotomy success. However, subgroup analyses (e.g. MTA vs. calcium hydroxide, MTA vs. Biodentine) showed overlapping confidence intervals and no evidence of publication bias, supporting consistency within these comparisons. No concerns about indirectness or industry sponsorship were noted.

#### Balance of benefit and harm

No serious adverse effects were reported in either systematic review [43, 68].

#### Ethical Considerations

No ethical challenges were observed.

#### Accessibility, affordability and equity issues

Due to the higher costs of HCSC compared to calcium hydroxide, the affordability of direct pulp capping or pulpotomy with HCSC may vary depending on the healthcare system, potentially leading to equity concerns [43].

#### Legal considerations

No legal considerations.

#### Economic considerations

Direct pulp capping or pulpotomy with HCSC is likely to be more costly, while lower risks of failure and pain may compensate for these costs long-term. Currently, economic data comparing both strategies is lacking.

#### Patient preferences and values

Unclear. Notably, certain HSCS may induce staining of the teeth, which is particularly relevant in anterior teeth. Dentists should be aware of and consider this in choosing the material to use while discussing the risk with the patient.

#### Applicability

Most studies were conducted in well-controlled university settings. Given that HSCS require particular training for proper application, applicability may be limited.

## Supplementary Information

Below is the link to the electronic supplementary material.

ESM 1 DOCX (34.3 KB)

## Data Availability

The data that support the findings of this study are available from the corresponding author upon reasonable request.

## Appendix: glossary of terms used in the recommendations

This glossary provides definitions of key terms and concepts used throughout these guideline recommendations. It is intended to support consistent understanding and application of the recommendations across different clinical and professional contexts.

- Caries
  The biochemical “caries process” occurs within the dysbiotic, stagnant oral plaque biofilm that resides on a dental hard tissue surface. The physical tissue damage that is caused by this process, when uncontrolled, on the underlying tooth structure is termed the “lesion of caries”. The lesion has several stages of physical progression. Nomenclature includes both the “carious lesion”,, the “caries lesion”, or – in this guideline – “caries”. Hence, we refer to caries when we mean both, the process and the lesion.
- Caries removal
  The process in which decayed infected (contaminated) and affected (demineralised) dental hard tissues (enamel and dentine) are separated from the healthy tissues, within a deep, or extremely deep cavitated caries. This process may include the use of physical cutting instruments (hand instrument excavation or rotary methods, drills and burs), air-abrasion technology or chemomechanical methods (using chemicals to soften diseased tissue which can be more simply abraded away with suitable hand instruments). A combination of the above could be used to remove discerning amounts of decayed dentine (see below).
- Deep caries
  Is that lesion that extends into the inner quarter of the dentine but retains a protective layer of hard or firm dentine over the pulp, reducing the risk of immediate exposure during the operative process. Depending on the radiographic technique, a radioopaque band of dentine is visible on the radiograph above the pulp.
- Extremely deep caries
  Is that lesion that penetrates the entire dentine thickness, making dental pulp exposure highly likely during operative treatment. Depending on the radiographic technique, no radioopaque band of dentine is visible on the radiograph above the pulp.
  Such lesion requires careful and time-sensitive management to preserve dental pulp vitality.
- Non-Selective Removal (excavation)
  Non-Selective removal/excavation to hard dentine (NSE), previously known as complete removal, applies a uniform standard for removing decayed tissue within the cavity, ensuring that only hard dentine remains throughout the cavity walls and pulpal floor (this feels scratchy to a sharp dental explorer/probe dragged across its surface). This approach eliminates all demineralized/affected dentine, even if bacteria-free.
- Post-operative pain.
  This is pain often of a few days duration, elicited from pulp nociceptors, triggered after deep caries removal or direct pulp tissue management. The pain can vary in intensity and duration and may be caused by direct stimulus to nerve fibres or odontoblasts, tubular fluid imbalance and movement, or in response to an acute inflammatory response to direct trauma or dental biomaterials interacting with viable pulp tissues.
- Pulp vitality (pulp sensibility)
  Dental pulp vitality is the ability fo the pulp tissue complex to maintain and or regenerate or “heal” itself and the overlying dentine. This ability is clinically assessed by detecting a response to nerve stimulation to temperature, electrical or direct mechanical pulp stimulation. These conventional procedures measuring the level of innervation, are more correctly termed *pulp sensibility tests*. Pulp vitality, another term commonly and historically used for the same outcome measure, strictly measures dynamic pulp blood flow (using laser Doppler flowmetry, which is not used in common clinical dental/endodontic practice). Other clinical investigations detect the pathological effects of pulp inflammation and infection, using radiography to identify bone resorption in the peri-apical region of the affected tooth (radiolucency/widening of the periodontal ligament space, peri-apical periodontitis). Clinical outcomes measures will include the interpretation of pain on palpation around the alveolar tissues adjacent to the root apices, presence of swelling and tenderness to percussion of the tooth in question. Pulp vitality has been used in this guideline.
- Pulpectomy
  A pulpectomy is the operative process of removing all of the pulp tissue within the pulp chamber and the complete root canal system, cleaning out all infected soft tissues. Technically, a root canal treatment adds the obturation of the disinfected root canal spaces with a suitable sealing material.
- Pulpotomy, partial
  Partial pulpotomy is defined as the removal of 2–3 mm of the exposed pulp tissues within the pulp chamber, after cavity preparation, followed by placement of a suitably biocompatible material to seal the pulp wound and permit an overlying sealed restoration. As only the superficial layer of infected and/or inflammed pulp tissue has been removed, this procedure allows the remaining vital coronal pulp tissue to regenerate and heal. The viability of this procedure and the amount of pulp removed is predicated on the level of inflammation within the superficial tissues and the ability of the pulpal blood flow to coagulate with a few minutes.
- Pulpotomy, full
  Full pulpotomy removes the all pulp tissues (including inflamed/diseased pulp tissue) from the coronal pulp chamber of the tooth leaving healthy pulp tissue within the root canal system, which is then dressed with a dental biomaterial that maintains vitality and promotes repair/healing.
- Selective removal (excavation)
  Selective removal/excavation (SE) retains firm or leathery dentine (feels sticky and scratchy to a sharp dental probe/explorer dragged across its surface) over the pulp while ensuring that peripheral dentine is hard. SE to soft dentin (formerly termed incomplete or partial removal) preserves soft carious tissues near the pulp to minimize exposure risk and stress while ensuring that peripheral dentine is prepared to a hard consistency for a secure restoration with an adhesive peripheral seal. In both cases, the definitive restoration is provided in the same visit.
- Stepwise removal (excavation)
  Stepwise removal/excavation (SW) is a staged approach to caries removal, completed over two separate visits. In the first stage, soft carious tissue is retained over the pulp, while the peripheral dentine is prepared to a hard consistency to allow for a sealed restoration. A provisional restoration is placed, designed to last up to 12 months or longer, to enable dentine and pulp changes to occur (Maltz et al. 2012a). This initial phase encourages reactionary dentine formation, promotes remineralization and decreases bacterial load. During re-entry in the second phase, the provisional restoration is removed and changes in lesion color and hardness are reassessed. Caries removal continues only until leathery dentine remains over the pulp. A definitive, sealed restoration is then placed.
- Vital Pulp Treatment (VPT)
  Vital pulp treatment (VPT) can be defined as treatment which aims to preserve and maintain the viability of pulp tissue that has been compromised, but not destroyed by extensive dental caries, dental trauma, restorative procedures or for iatrogenic reasons. The term covers a range of endodontic procedures, for example direct pulp capping/protection, partial pulpotomy and full pulpotomy.

## Additional Information

This article is simultaneously published in Clinical Oral Investigations 10.1007/s00784-025-06727-1, International Endodontic Journal 10.1111/iej.70132 and Caries Research10.1159/000551659. This article was jointly published in *Clinical Oral Investigations, International Endodontic Journal and Caries Research*. The articles are identical except for minor stylistic and spelling differences in keeping with each journal’s style. Either citation can be used when citing this article.
