# Supplementary material for: Deep caries management: EFCD-ESE-ORCA S3-level clinical practice guideline
Source: Clin Oral Investig. 2026 Apr 22;30(5):186. doi: 10.1007/s00784-025-06727-1 (PMC13099699; doi:10.1007/s00784-025-06727-1)
Supplement: Supplementary file 1 — DOCX (34.3 KB) [file 784_2025_6727_MOESM1_ESM.docx]

**Supporting Information**

Supplementary Table 1: Key stakeholders contacted

| **Invited society/organisation** | **Acronym** | **Answer** | **Representative** |
| --- | --- | --- | --- |
| Association for Dental Education in Europe | ADEE | Agreed | Barry Quinn |
| BundesArbeitsGemeinschaft der Patientenstellen und -Initiativen | BAGP | No answer | - |
| Council of European Chief Dental Officers | CECDO | No answer | - |
| Council of European Dentists | CED | Declined | - |
| German Society of Dentistry and Oral Medicine | DGZMK | Agreed | Christian Gerhardt |
| European Association of Dental Public Health | EADPH | Agreed | Lina Stangvaltaite-Mouhat |
| European Association of Oral Medicine | EAOM | Agreed | Avijit Banerjee |
| European Academy of Pediatric Dentistry | EAPD | Declined | - |
| European College of Gerodontology | ECG | Agreed | Martina Hayes |
| European Dental Hygienist Federation | EDHF | Agreed | Simone Ruzario |
| European Dental Students Association | EDSA | Agreed | Saulė Skinkytė |
| The European Forum for Primary Care | EFPC | No answer | - |
| European Prosthodontic Association | EPA | No answer | - |
| European Regional Organization of the FDI | ERO | No answer | - |
| The European Patients’ Academy | EUPATI | Agreed | Shalya Anand |
| International Association of Dental Traumatology | IADT | Agreed | Anne O'Connell |
| Pan-European Region – International Association for Dental Research | PER-IADR | Agreed | Laura Ceballos |

Supplementary Table 2: Guideline panel

| **Scientific society or organization** | **Delegate(s)** |
| --- | --- |
| *Responsible scientific societies* | |
| European Federation of Conservative Dentistry | Falk Schwendicke |
| European Society of Endodontology | Hal Duncan, Lars Bjørndal |
| Organization for Caries Research | Aylin Baysan, Klaus Neuhaus |
| Deutsche Gesellschaft fuer Zahnerhaltung | Sebastian Paris |
|  | Guideline coordinators: Esra Kosan, Helena Dujic  Methodologist: Ina Kopp |
| *Scientific societies involved in the guideline development* | |
| Association for Dental Education in Europe | Barry Quinn |
| German Society of Dentistry and Oral Medicine | Christian Gerhardt |
| European Association of Dental Public Health | Lina Stangvaltaite-Mouhat |
| European Association of Oral Medicine | Avijit Banerjee |
| European College of Gerodontology | Martina Hayes |
| International Association of Dental Traumatology | Anne O'Connell |
| International Association for Dental Research, Pan-European Region | Laura Ceballos |
| *Other organizations* | |
| European Dental Hygienist Federation | Simone Ruzario |
| European Dental Students Association | Saulė Skinkytė |
| *Patient representatives* | |
| The European Patients’ Academy | Shalya Anand |

Supplementary Table 3 Declaration of interests and handling of conflicts of interest. The following section presents a tabular summary of the declarations of interest, along with the results of the conflict of interest assessment and the measures decided upon by the guideline group after discussion of the individual cases. These measures were implemented as part of the consensus conference.

|  | Activity as a consultant and/or expert | Participation in a scientific advisory board | Paid lecture or training activity | Paid authorship or co-authorship | Research projects/  Conducting clinical studies | Ownership  interests (patent, copyright rights, share ownership) | Indirect interests | Guideline topics affected by COI,  Classification with regard to relevance,  Consequence |
| --- | --- | --- | --- | --- | --- | --- | --- | --- |
| Anand, Shalya | None declared | None declared | None declared | None declared | None declared | None declared | Member: Chair of the Oral Health Literacy Division, International Health Literacy Association  Scientific activity: Oral health in geriatric oncology  Clinical activity: Oral health literacy  Involvement in education/training: —  Personal relationship: None | COI: none |
| Banerjee, Avijit | vVardis | Prevolution Health | Oral B, Septodont, GC | A Clinical Guide to Advanced Minimum Intervention Restorative Dentistry, Elsevier.    Odell's Clinical Problem Solving in Dentistry ed 4. Elsevier | NIHR HTA / Dundee | None declared | Member: President-elect, British Society of Oral and Dental Research, Honorary Consultant Advisor, Office of the Chief Dental Officer, England, Member, Health and Science Committee, British Dental Association  Scientific activity: 180 publications related to dental caries imaging, detection, diagnosis, operative treatment, dental biomaterials, clinical trial implementation, and policy  Clinical activity: Restorative management of oral cancer patients; periodontal disease management  Involvement in education/training: Programme Director, MSc in Advanced Minimum Intervention Restorative Dentistry, King’s College London  Personal relationship: N/A | COI: moderate: Possible conflict of interest regarding materials used in deep caries management. Should abstain from voting on recommendations regarding materials used in deep caries management. |
| Baysan, Aylin | N/A | N/A | N/A | N/A | N/A | N/A | Member: N/A  Scientific activity: N/A  Clinical activity: N/A  Involvement in education/training: N/A  Personal relationship: N/A | COI: none |
| Bjørndal, Lars | Dental Knowledge center. (About the Dental Knowledge Center, Denmark) | Editorial board of Dental journals ( (IEJ) | Several international meetings as an invited speaker but never funded by a Firm/ enterprise | Several papers and chapters about this topic | AI in Denistry.  Copenhagen Univeristy and Department of odontology | None declared | Member: Participates as an expert in the IAPD Global Consensus on Pulp Therapies in Primary and Permanent Teeth (no financial involvement; ends Summer 2025)  Scientific activity: Artificial intelligence in dentistry; aspects of deep caries  Clinical activity: Endodontic vital pulp therapy; operative therapy; cariology  Involvement in education/training: Professor in Cariology and Endodontics, University of Copenhagen (KU), Denmark | COI: none |
| Ceballos, Laura | None declared | COLGATE PALMOLIVE ESPAÑA S.A., COLGATE PALMOLIVE ESPAÑA S.A. | COLGATE PALMOLIVE ESPAÑA S.A., COLGATE PALMOLIVE ESPAÑA S.A., COLGATE PALMOLIVE ESPAÑA S.A., COLGATE PALMOLIVE ESPAÑA S.A., Procter Gamble Spain and Portugal | None declared | ALIGN TECHNOLOGY SWITZERLAND GMBH | Univeridad Rey Juan Carlos-Universidad de Granada | Member: Board Member, Continental European Division of the International Association for Dental Research (CED-IADR) Board Member, European Federation of Conservative Dentistry (EFCD) Member, Spanish Society of Conservative and Esthetic Dentistry (SEOC)  Scientific activity: Dental bleaching; management of deep caries lesions; adhesion to dentin and various restorative materials; development and characterization of new dental materials | COI: none |
| Dujic, Helena | None declared | None declared | None declared | None declared | None declared | None declared | Member: None  Scientific activity: Artificial intelligence and dental diagnostics; guideline development on the management of genetic dental disorders  Clinical activity: Preventive care; conservative dentistry  Involvement in education/training: None  Personal relationship: None | COI: none |
| Duncan, Henry (Hal) Fergus | None declared | None declared | None declared | None declared | None declared | None declared | Member: President, European Society of Endodontology  Scientific activity: Editor-in-Chief, International Endodontic Journal | COI: none |
| Gernhardt, Christian R. | Court-appointed expert in cooperation with various courts | Zahnärztekammer Sachsen-Anhalt  Förderpreis der Kammer Wissenschaftlicher Beirat, 1. Vorsitzender der Akademie Praxis und Wissenschaft der DGZMK e.V. | Professional involvement / affiliations: Academy of Practice and Science (APW)  Dental Chambers: Dental Chamber of Saxony-Anhalt, Dental Chamber of North Rhine, Dental Chamber of Schleswig-Holstein, Dental Chamber of Baden-Württemberg, Others  Professional Societies: German Society for Endodontology and Dental Traumatology (DGET), German Society for Conservative Dentistry (DGZ), German Society for Implantology (DGI)  Industry Collaborations: Voco GmbH, Cuxhaven, Bego GmbH, Bremen | None declared | Voco, Cuxhaven, Komet | None declared | Member: Dental Chamber of Saxony-Anhalt: Member and Board Member as Continuing Education Officer, German Society for Endodontology and Dental Traumatology (DGET): Member and Board Member (President until 2019, Board Member until 2023), German Society for Conservative Dentistry (DGZ): Member and Board Member until 11/2019, German Society of Dentistry and Oral Medicine (DGZMK): Board Member since 09/2024, German Society of Implantology (DGI): Member, Society for Dentistry, Oral and Maxillofacial Medicine at Martin Luther University Halle-Wittenberg: Member and Chair, Academy of Practice and Science of the DGZMK (APW): Deputy Chair and since 09/2024 Chairperson  Scientific activity: Conservative dentistry; cariology; endodontics; restorative dentistry; dentin hypersensitivity  Clinical activity: Conservative dentistry; periodontology; pediatric dentistry  Involvement in education/training: Academy of Practice and Science (APW): Deputy Chair and, since 09/2024, Chairperson – responsible for developing continuing education programs, Dental Chamber of Saxony-Anhalt: Continuing Education Officer – responsible for designing continuing education programs  Personal relationship: Dr. Juliane Gernhardt, practicing in a private clinic in Halle | COI: none |
| Hayes, Martina | None declared | None declared | None declared | None declared | Cystic Fibrosis Ireland, Health Research Board (or Ireland) | None declared | Member: European College of Gerodontology (ECG)  Involvement in education/training: Full-time faculty member at University College Cork | COI: none |
| Herbst, Chantal Sophie | None declared | None declared | None declared | None declared | None declared | None declared | None declared | COI: none |
| Herbst, Sascha | None declared | None declared | Septodont | None declared | None declared | None declared | Member: Member and active participant in the German Society for Endodontology and Dental Traumatology (DGET e.V.) | COI: moderate; No mandate in this guideline. |
| Hildebrand, Hauke | None declared | None declared | None declared | None declared | None declared | None declared | Member: Swiss Society for Preventive, Restorative and Aesthetic Dentistry (SSPRE) – Member, Swiss Society of Endodontology (SSE) – Member, German Society for Endodontology and Dental Traumatology (DGET) – Member, German Society of Dentistry and Oral Medicine (DGZMK) – Member  Scientific activity: Endodontics  Louzada LM, Hildebrand H, Neuhaus KW, Duncan HF. The effectiveness of partial pulpotomy compared with full pulpotomy in managing deep caries in vital permanent teeth with a diagnosis of non-traumatic pulpitis. Int Endod J. 2025 Jan;58(1):37-54. doi: 10.1111/iej.14149. Epub 2024 Sep 12. PMID: 39264795; PMCID: PMC11629071.  Clinical activity: Endodontics; conservative dentistry  Involvement in education/training: No  Personal relationship: No | COI: none |
| Kopp, Ina | German Accreditation Body (DAkkS), European Federation of Periodontology, British Society for Periodontology, European Society of Endotontology (ESE), European Society for Contact Dermatitis (ESCD), European Academy for Childhood Disability (EACD) | Agency for Quality in Medicine (ÄZQ) (a non proft GIN member organisation) | EBM Frankfurt, Working Group at the Institute for Family Medicine, Goethe-University Frankfurt, German Society for Paediatric Infectiology, European Business School (EBS) Wiesbaden, European Association of Dental Implantologists (BDIZ) | None declared | German Cancer Aid Foundation, German Ministry of Health (BMG) , German Federal Joint Committee independent Funding Programme for Clinical Practice Guidelines developed under the auspices of AWMF member societies, German Ministry for Education and Research (BMBF), German Ministry for Education and Research (BMBF), German Federal Joint Committee , German Federal Joint Committee | None declared | Member: AWMF Representative on the Board of Trustees, IQTIG (German Institute for Quality and Transparency in Health Care – a non-profit governmental institution), AWMF Representative on the Board of Trustees, IQWiG (German Institute for Quality and Efficiency in Health Care – a non-profit governmental institution), Honorary Member, German Society for Breast Health, Member, German Network for Evidence-Based Medicine (DNEbM); German Society of Surgery (DGCH), Member, Steering Committee for the Guideline Program in Oncology (German Cancer Society, German Cancer Aid, and AWMF), Member, Advisory Board for the Program "National Disease Management Guidelines" (German Cancer Society and AWMF; term until Dec 31, 2024), Deputy Chair, Standing Commission on Guidelines of the AWMF, Primary Contact on behalf of AWMF in the Guidelines International Network (GIN), Trustee, Guidelines International Network (GIN)  Scientific activity: Guidelines; health services research; digitalisation  Clinical activity: Guidelines; health services research; digitalisation  Involvement in education/training: AWMF Curriculum for Guideline Developers and Guideline Advisors, Reviewer, GIN–McMaster INGUIDE Program  Personal relationship: None | COI: none |
| Kosan, Deniz | None declared | None declared | None declared | None declared | Charité | None declared | None declared | COI: none |
| Kosan, Esra | None declared | None declared | None declared | Zahnarzt Wirtschaft Praxis (Dental Economics and Practice), Zahnärztliche Mitteilungen (German Dental Journal), Kieferorthopädische Nachrichten (Orthodontic News)  German Society for Conservative Dentistry (DGZ) – involved in guideline development | Charité | None declared | Member: Free Association of German Dentists (Freier Verband Deutscher Zahnärzte), Berlin Dental Chamber (Zahnärztekammer Berlin), Pan-European Region of the International Association for Dental Research (PER-IADR) – Young Ambassador  Scientific activity: Artificial intelligence in dentistry; caries prevention during orthodontic treatment; dental trauma; in vitro studies on root posts; dental adhesives; composite restorations; guideline development; dental radiology  Clinical activity: Radiology and diagnostics; dental trauma; endodontics; restorative dentistry; periodontology; oral surgery  Involvement in education/training: none  Personal relationship: none | COI: none |
| Marré, Birgit | Institute of Forensic Medicine at TU Dresden | None declared | None declared | None declared | None declared | None declared | Member: German Society of Dentistry and Oral Medicine (DGZMK), Working Group for Functional Diagnostics and Therapy (AKFOS), Working Group for Further Dental Education (AKWLZ), German Society of Dental, Oral and Craniomandibular Sciences (DGFDT), German Society for Prosthetic Dentistry and Biomaterials (DGPro) – Member  Scientific activity: None  Clinical activity: Prosthetic dentistry  Involvement in education/training: None  Personal relationship: None | COI: none |
| Mendes Louzada, Lidiane | None declared | None declared | None declared | None declared | None declared | None declared | Member: None  Scientific activity: None  Clinical activity: None  Involvement in education/training: None  Personal relationship: None | COI: none |
| Neuhaus, Klaus | Lutz Zürrer Stiftung | Toothfriendly International, ORCA | diverse | Quintessenz Verlag | None declared | None declared | Scientific activity: Caries diagnostics; root canal irrigation  Clinical activity: General dentistry; pediatric dentistry | COI: none |
| O´Connell, Anne | International Association of Paediatric Dentistry\  International Association of Dental Traumatology | None declared | None declared | IAPD | None declared | None declared | Member: Caries Trauma Paediatrics, Scientific activity: Author of trauma prevention guidelines published on caries diagnosis, consensus bn pulp response | COI: none |
| Paris, Sebastian | MARS | Stiftung Innovative Zahnmedizin (Foundation for Innovative Dentistry) | Member / Professional Involvement:  DMG (Dental Material Company)  Stiftung Innovative Zahnmedizin (Foundation for Innovative Dentistry)  Kulzer (Dental manufacturer)  Various Dental Chambers (Zahnärztekammern)  Continuing education institutes  Professional dental societies  University of Greifswald | Quintessenz | None declared | Charité - Lizenzierung DMG | Member: Board Member and current President, German Society for Conservative Dentistry (DGZ), Board Member, European Federation of Conservative Dentistry (EFCD)  Scientific activity: Caries prevention and therapy; general restorative dentistry  Clinical activity: Practicing dentist  Involvement in education/training: Clinical teaching at Charité – Universitätsmedizin Berlin | COI: none |
| Pitchika, Vinay | None declared | None declared | None declared | None declared | None declared | None declared | Member: International Association for Dental Research (IADR), German Society of Dentistry and Oral Medicine (DGZMK), German Society of Periodontology (DGParo)  Scientific activity: Periodontology; dental epidemiology  Clinical activity: Not applicable  Involvement in education/training: Not applicable  Personal relationship: Not applicable | COI: none |
| Quinn, Barry | Nil | None declared | None declared | None declared | None declared | None declared | Member: Association for Dental Education in Europe (ADEE)  Scientific activity: International Association for Dental Research (IADR)  Clinical activity: Royal Society of Medicine  Involvement in education/training: Haptic VR Thinkers | COI: none |
| Ramezanzade, Shaqayeq | None declared | None declared | None declared | None declared | None declared | None declared | None declared | COI: none |
| Ruzario, Simone | Proctor Gamble (Oral B Employee), Kenvue/SEPA | None declared | None declared | None declared | None declared | None declared | Member: British Society of Dental Hygiene and Therapy, General Dental Council  Scientific activity: Scottish Dental Clinical Effectiveness Programme (SDCEP) – Short Life Working Group, Oral Health Research Incubator | COI: none |
| Prof. Dr. Schlüter, Nadine | None declared | None declared | None declared | None declared | None declared | None declared | None declared | None |
| Schmidt, Wiebke | - | - | - | - | - | - | Member: German Society of Dentistry and Oral Medicine (DGZMK), German Society for Prosthetic Dentistry and Biomaterials (DGPro), German Society for Dental Materials Science (DGZMB)  Scientific activity: None Clinical activity: None Involvement in education/training: None Personal relationship: None | COI: none |
| Schwendicke, Falk | None | Dentsply | Solventum, DMG, Septodont | None | None | None | Member: Guideline Coordinator, German Society for Conservative Dentistry (DGZ)  Scientific activity: Habilitation on deep caries | COI: none |
| Skinkyte, Saule | none | none | none | none | none | none | Member: European Dental Students’ Association (EDSA)  Scientific activity: None Clinical activity: None Involvement in education/training: None Personal relationship: None | COI: none |
| Stangvaltaite-Mouhat, Lina | none | none | Invited speaker at conference organized by the Lithuanian Dental Chamber | none | none | none | Member: None  Scientific activity: Deep caries: epidemiology; management preferences among dentists and dental students; clinical outcomes of vital pulp therapies  Clinical activity: None  Involvement in education/training: None  Personal relationship: None | COI: none |
| Stratigaki, Erini | None declared | None declared | None declared | None declared | None declared | None declared | None declared | None |
| Sturm, Richard | none | none | Philipp-Pfaff-Institute | none | none | none | Member: DGZMK, DGZ, DGET  Scientific activity: Post-endodontic restoration  Scientific activity: Endodontics | COI: none |
| Volgenant, Catherine | none | none | none | none | none | none | none | COI: none |
